# Supplementary material for: Autoinhibitory sterol sulfates mediate programmed cell death in a bloom-forming marine diatom
Source: Nat Commun. 2017 Nov 3;8:1292. doi: 10.1038/s41467-017-01300-1 (PMC5670183; doi:10.1038/s41467-017-01300-1)
Supplement: Supplementary file 1 — Supplementary Information [file 41467_2017_1300_MOESM1_ESM.pdf]

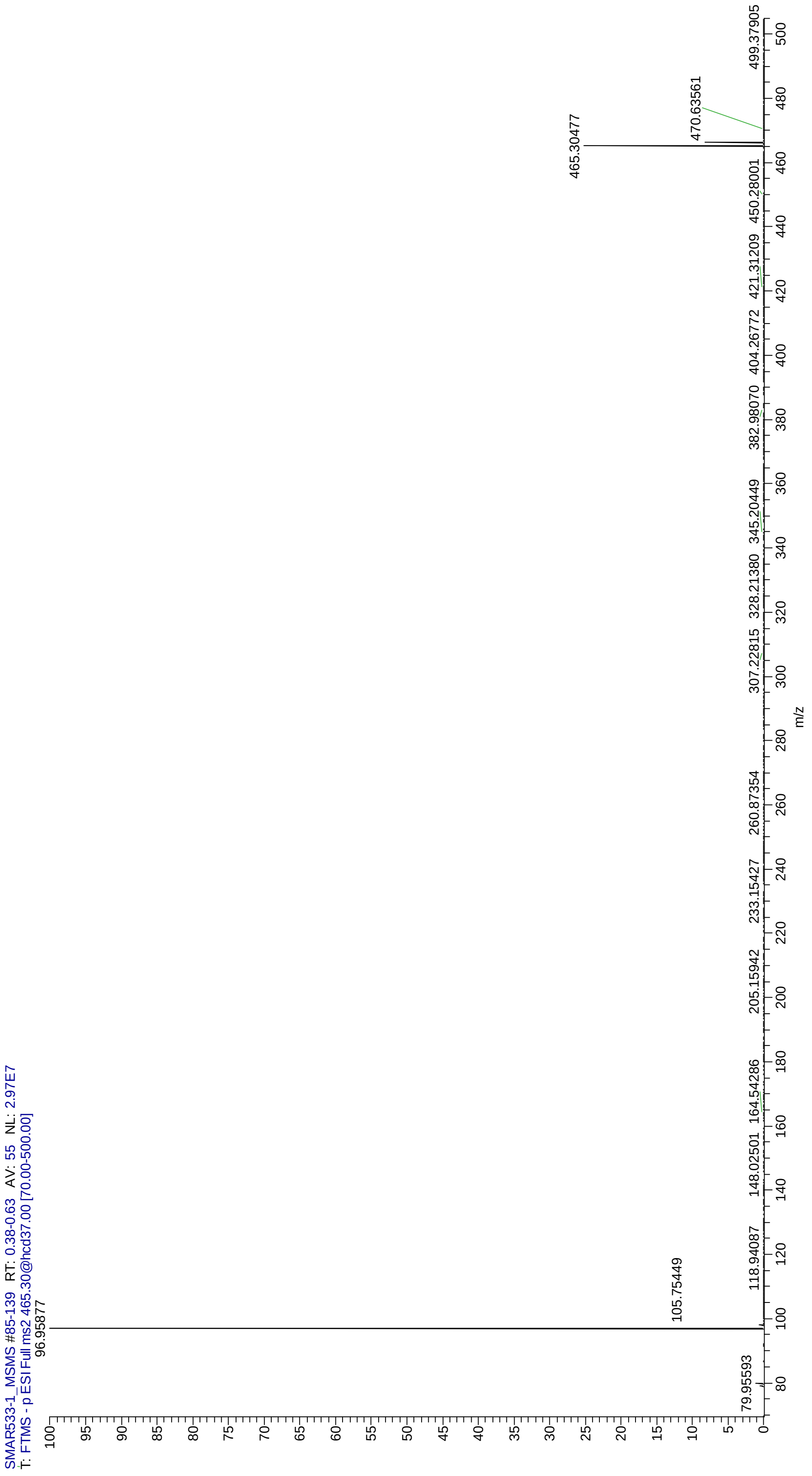

Supplementary Figure 1. High resolution ESI$^-$ MS/MS spectrum of cholesterol sulfate (**CHOS**).

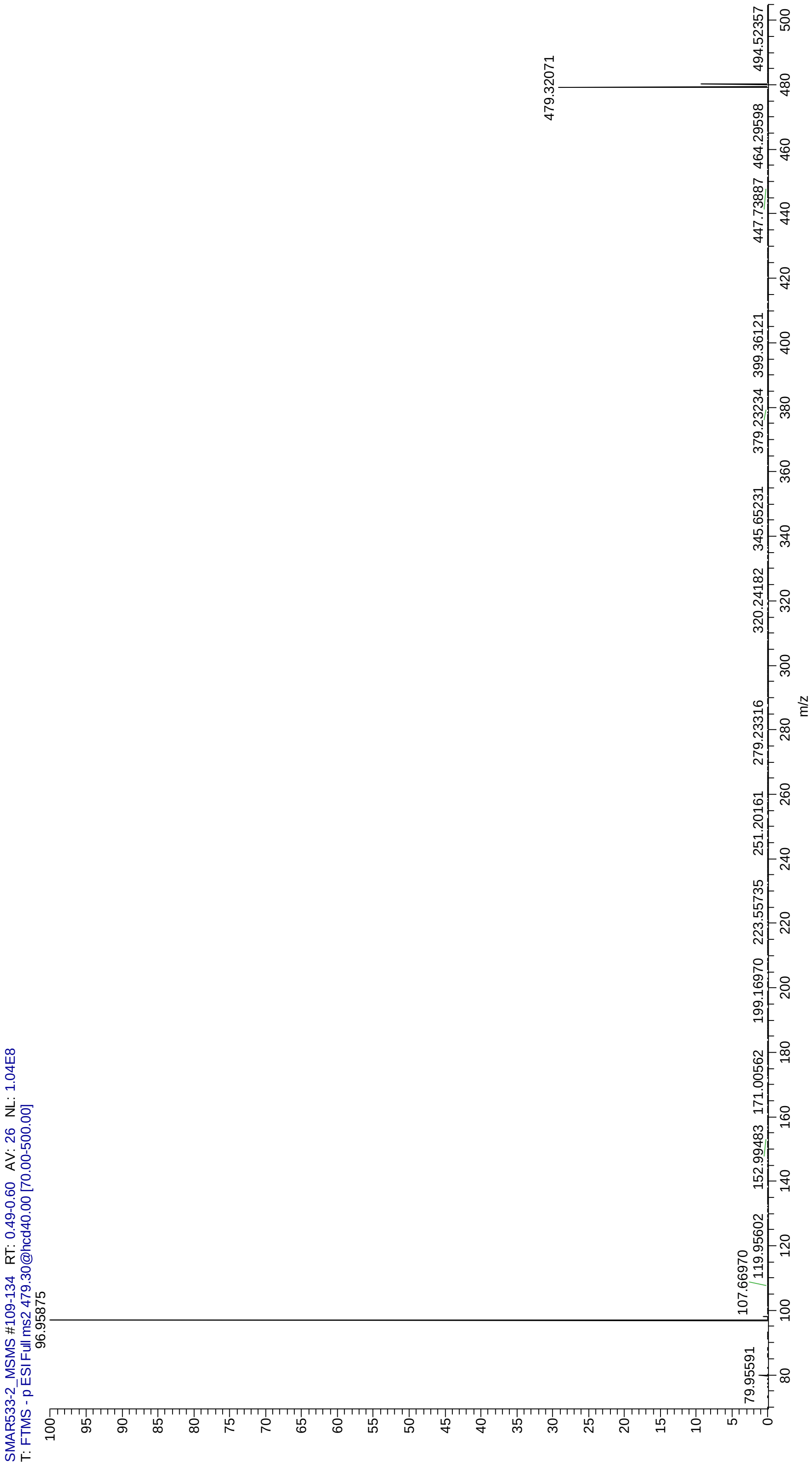

Supplementary Figure 2. High resolution ESI^{-} MS/MS spectrum of dihydro-brassicasterol sulfate (**DHBS**).

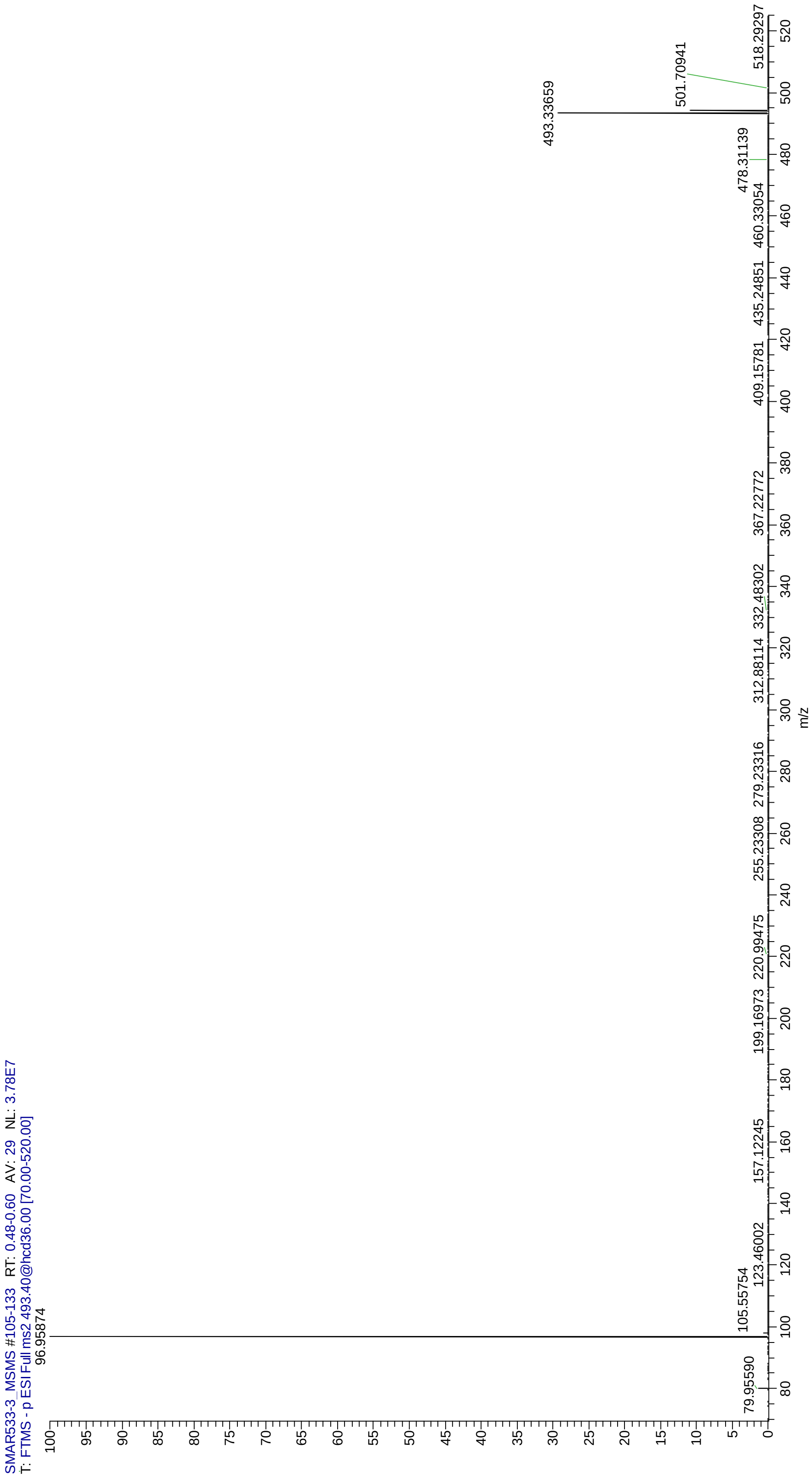

Supplementary Figure 3. High resolution ESI$^-$ MS/MS spectrum of β-sitosterol sulfate (**βSITS**).

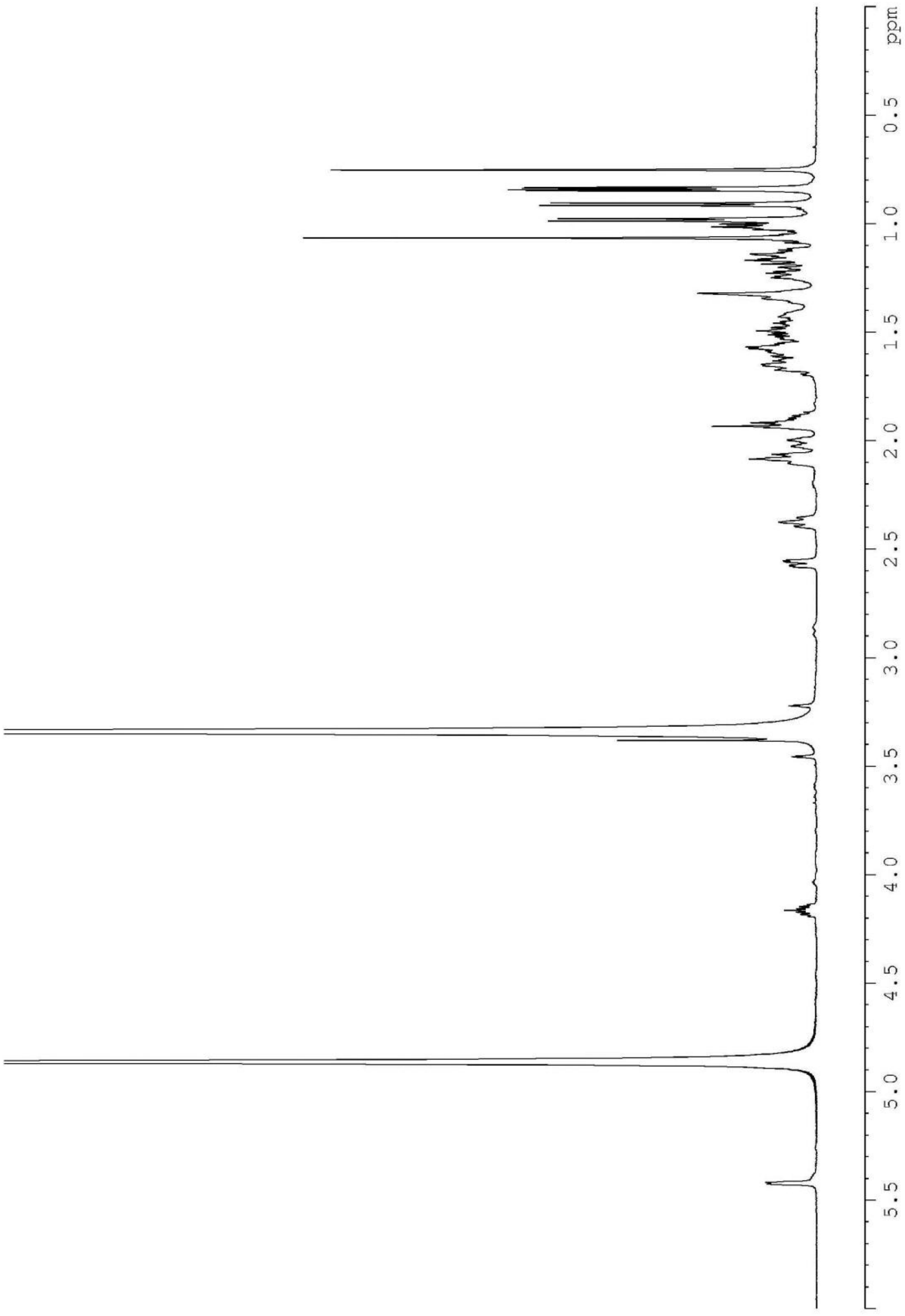

Supplementary Figure 4. ^{1}H NMR spectrum (CD_3OD, 600 MHz) of dihydrobrassicasterol sulfate (**DHBS**) from *S.marinoi* extracts.

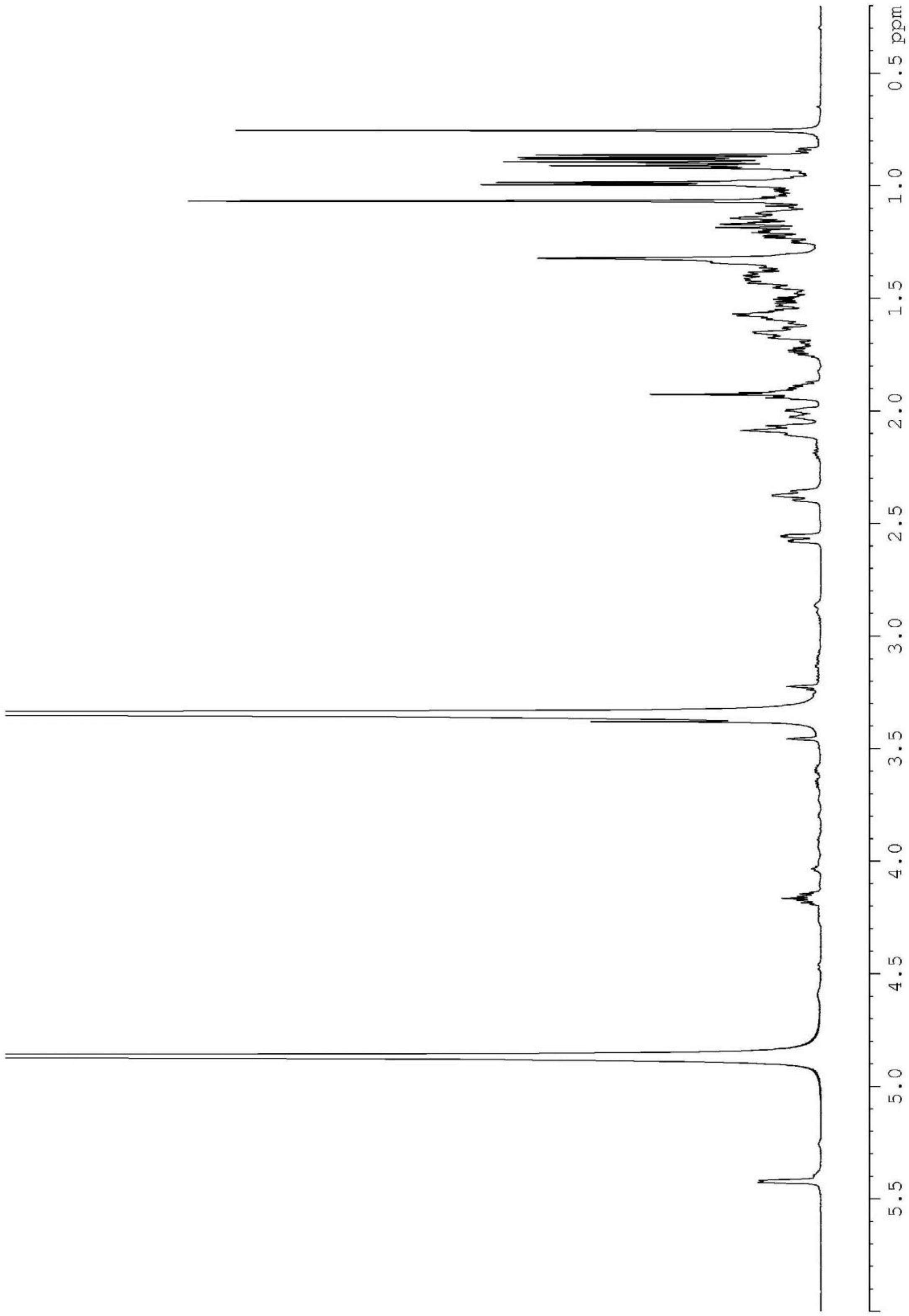

Supplementary Figure 5. ^{1}H NMR spectrum (CD_3OD, 600 MHz) of β-sitosterol sulfate (**βSITS**) from *S.marinoi* extracts.

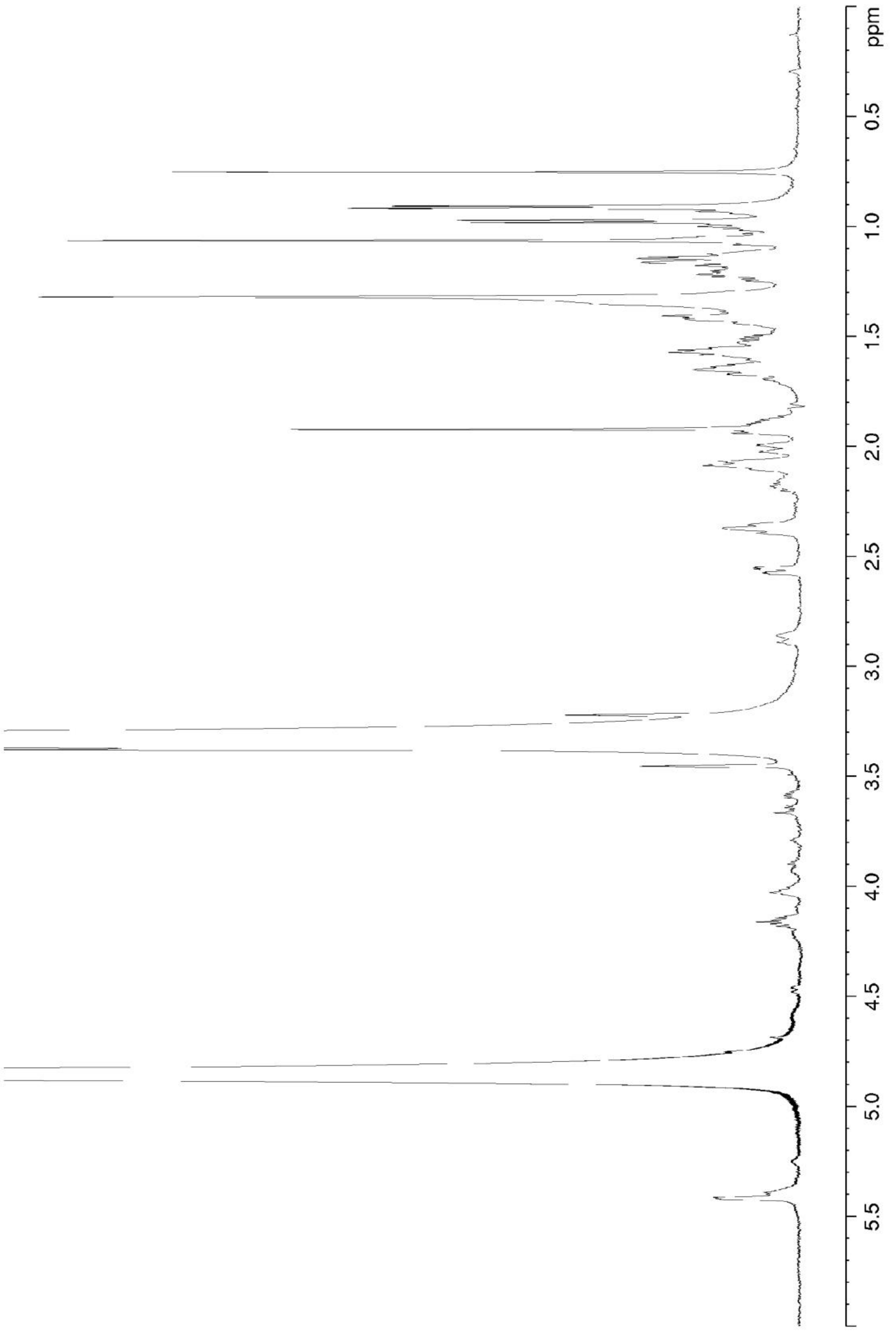

Supplementary Figure 6. ^{1}H NMR spectrum (CD_3OD, 600 MHz) of cholesterol sulfate (**CHOS**) from *S.marinoi* extracts.

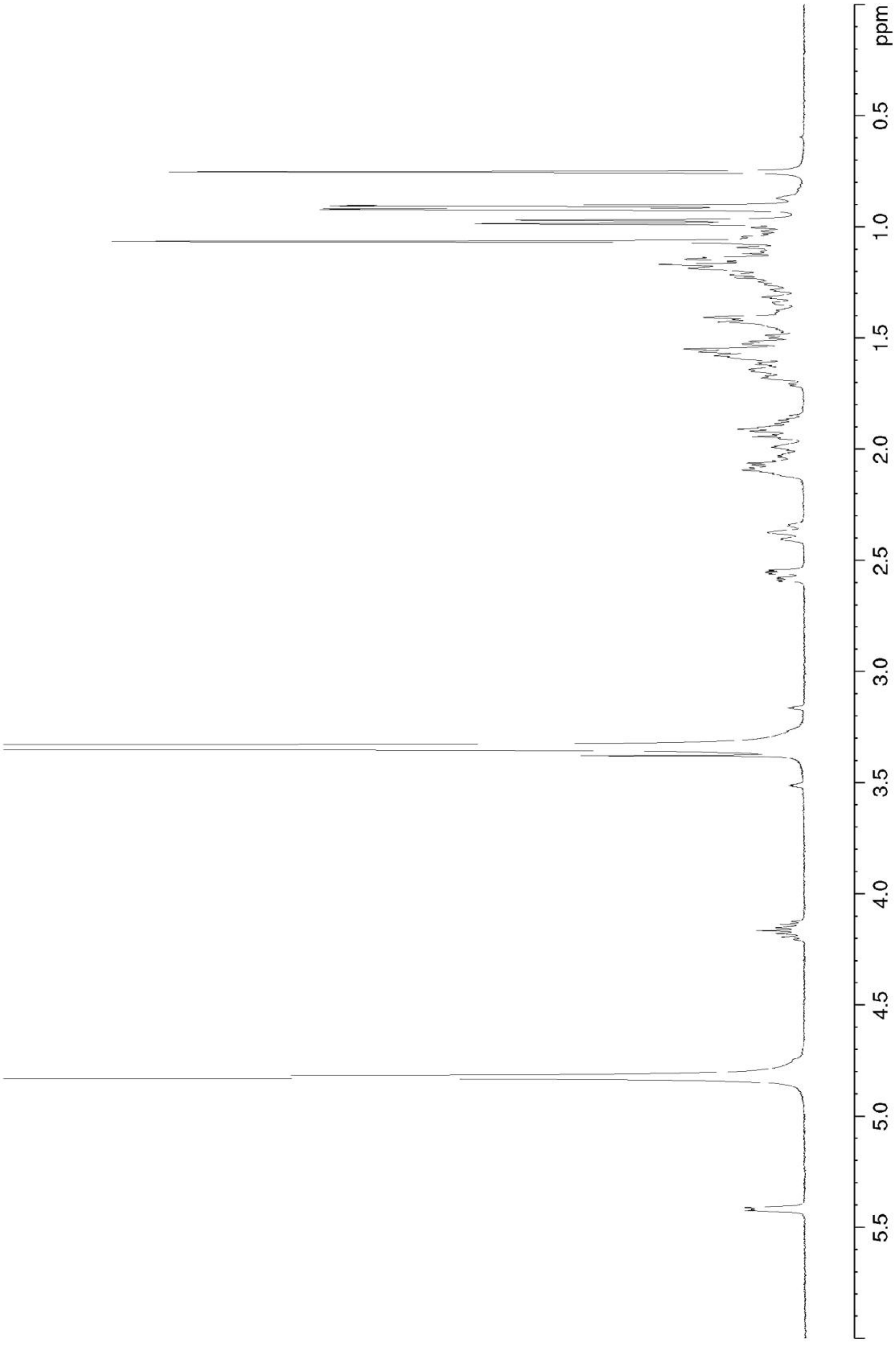

Supplementary Figure 7. ^{1}H NMR spectrum (CD_3OD, 400 MHz) of an authentic standard of cholesterol sulfate (**CHOS**).

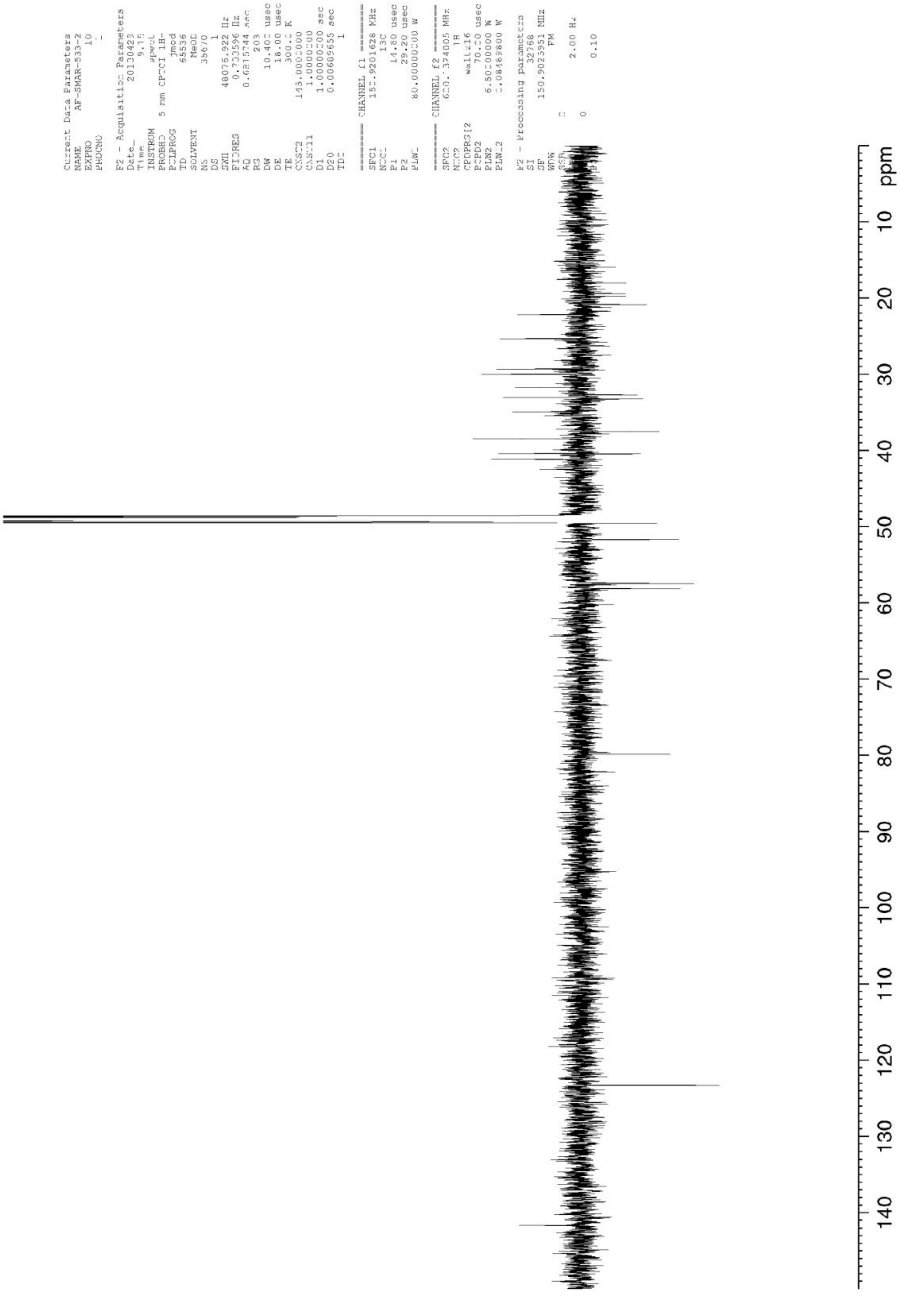

Supplementary Figure 8. ^{13}C-JMOD spectrum (CD_3OD, 600 MHz) of dihydrobrassicasterol sulfate (**DHBS**) from *S.marinoi* extracts.

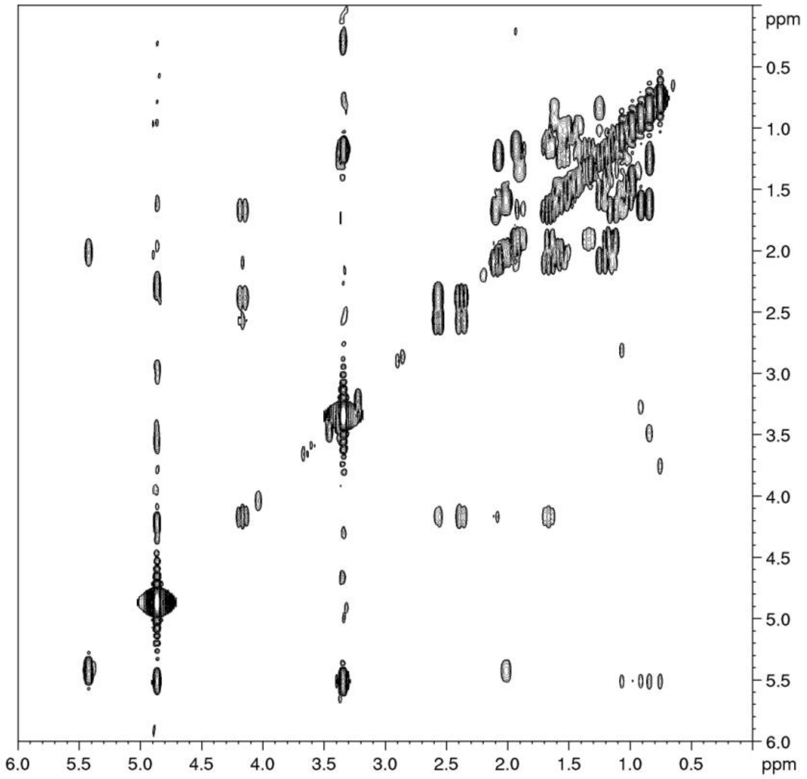

Supplementary Figure 9. ^{1}H-^{1}H COSY spectrum (CD_3OD, 600 MHz) of dihydrobrassicasterol sulfate (**DHBS**) from *S.marinoi* extracts.

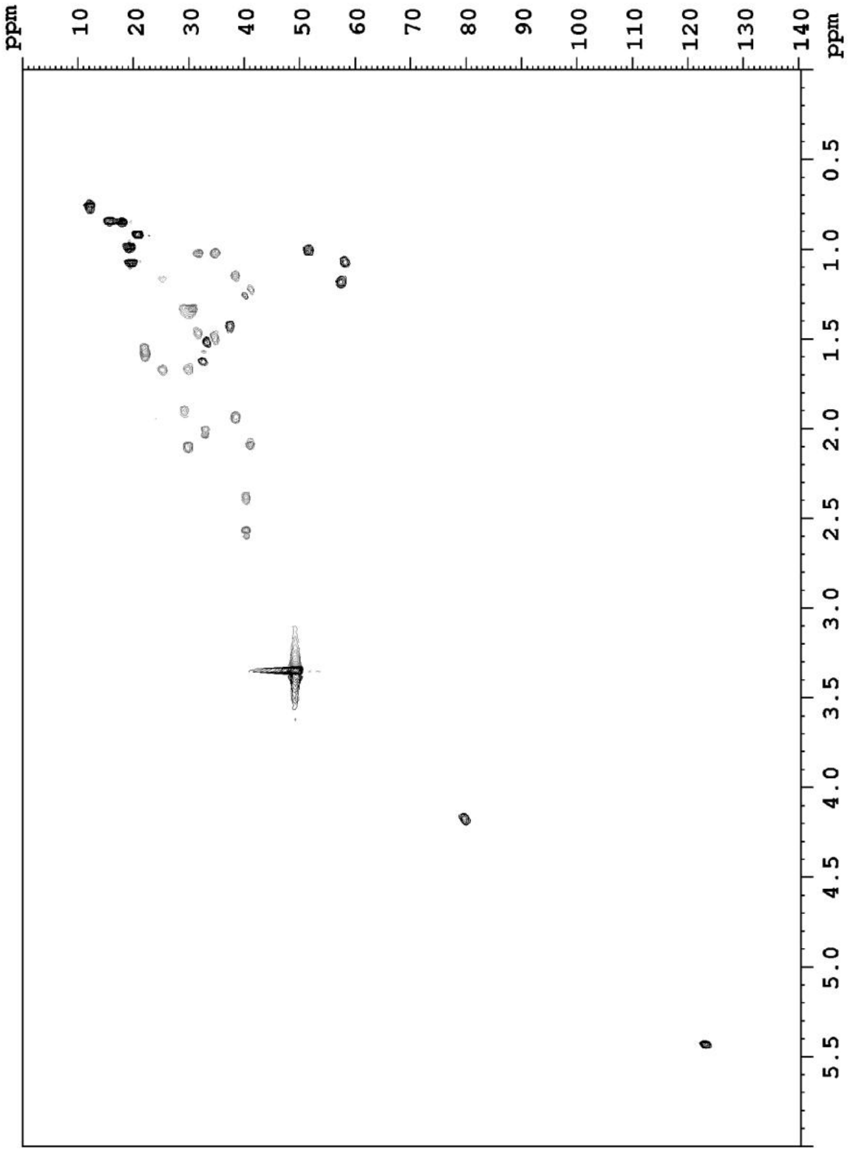

Supplementary Figure 10. HSQC spectrum (CD_3OD, 600 MHz) of dihydrobrassicasterol sulfate (**DHBS**) from *S.marinoi* extracts.

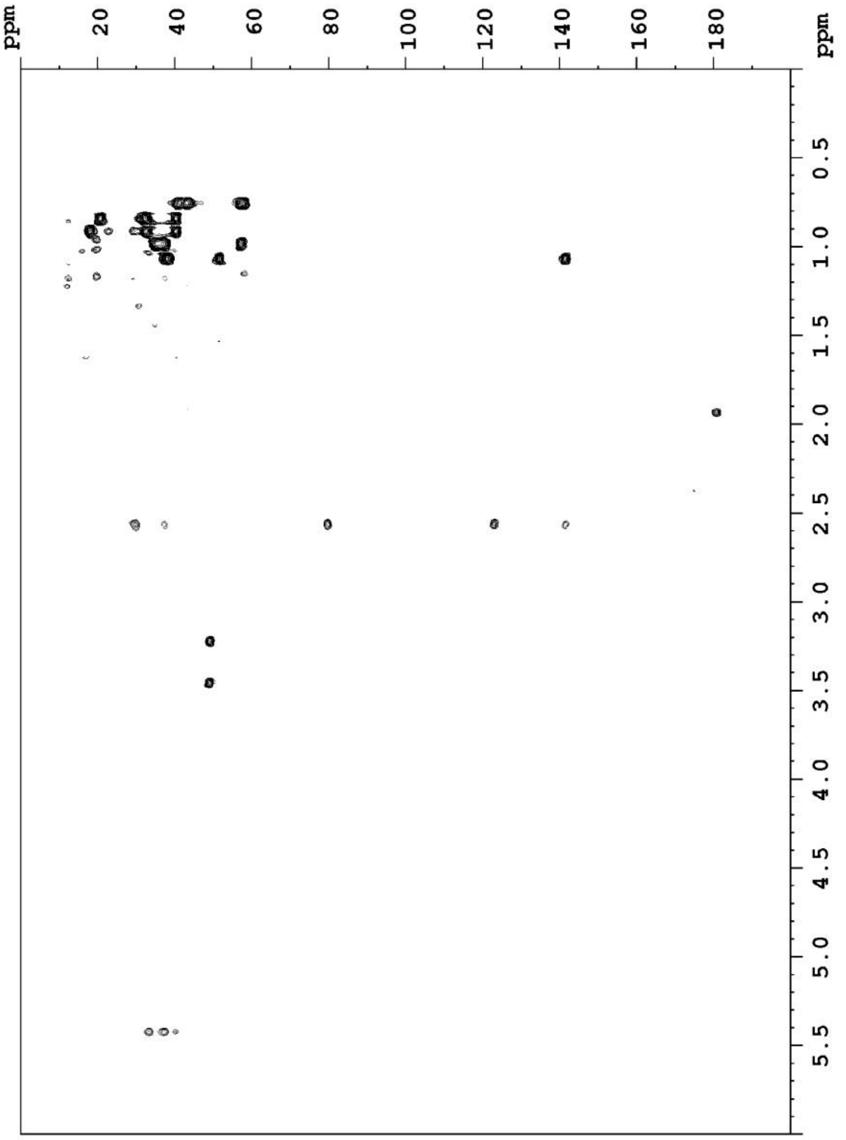

Supplementary Figure 11. HMBC spectrum (CD_3OD, 600 MHz) of dihydrobrassicasterol sulfate (**DHBS**) from *S.marinoi* extracts.

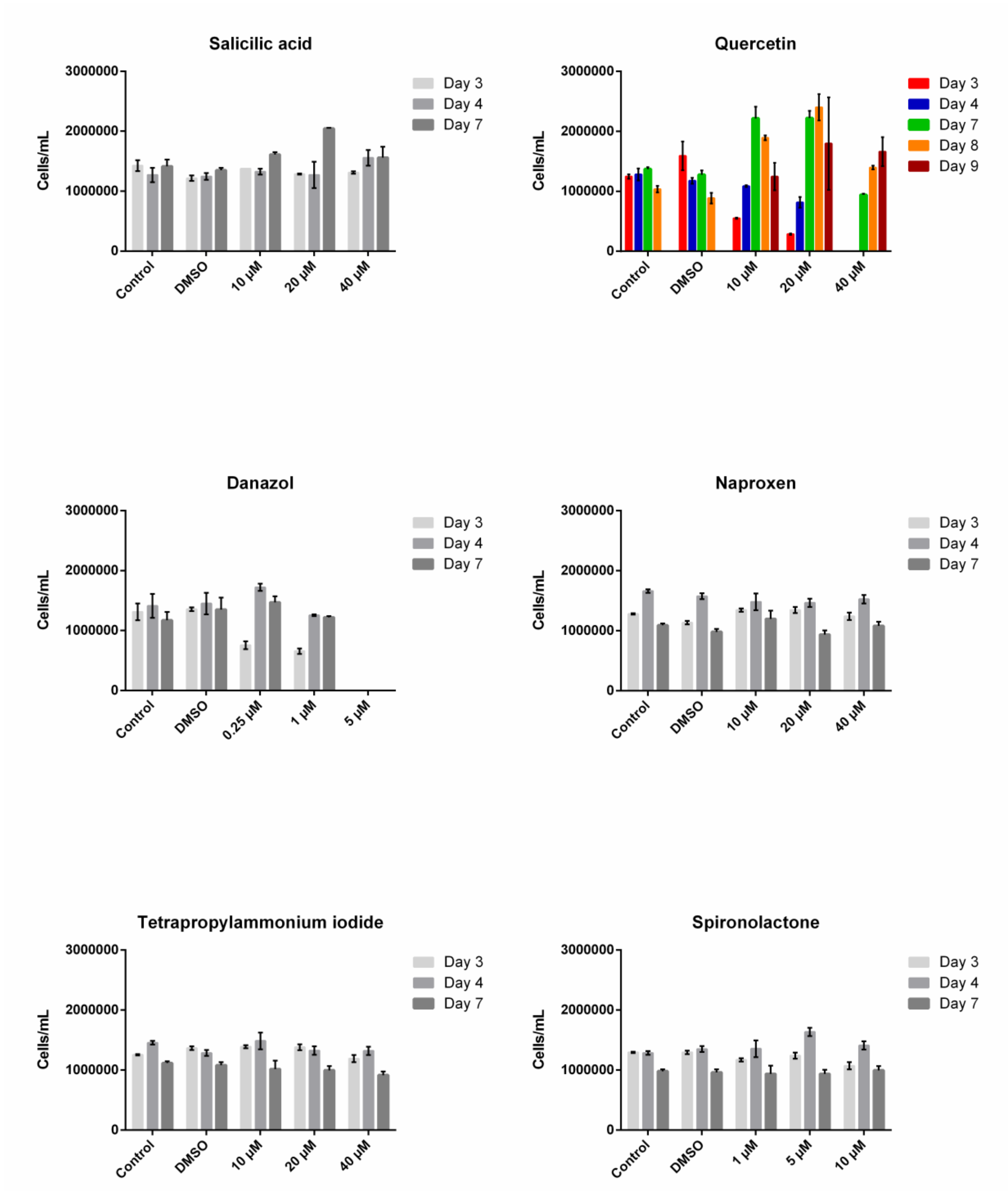

Supplementary Figure 12. Results of the screening of SULT inhibitors on *S. marinoi* cells. Data are expressed as cellular concentration (cell mL^{-1}) at day 3,4 and 7 at different concentrations of compound tested, compared to cells growth under standard conditions (control) and cells growth with 10 µL of DMSO (solvent used to dissolve test compounds). While the other tested compounds have a toxic effect or none effect on *S. marinoi* growth, quercetin showed a proliferative effect in the concentration range 10-20 µM. For each test we reported only the results for the concentration range that didn't' negatively affect vitality. Clomiphene was also tested, but it resulted toxic in the whole concentration range tested (0.25- 40 µM) with the 100% of cell mortality already in 24 hours. Data are means ± s.d.

A

B

C

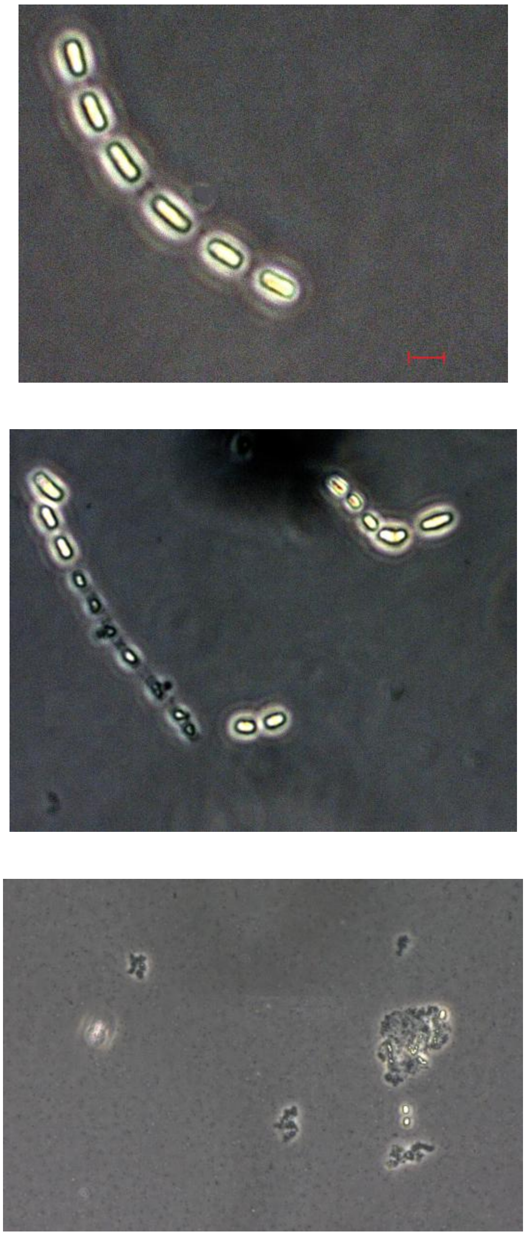

Supplementary Figure 13. Representative images of *S. marinoi* cells treated with extracts from end of log phase (**A**), early (**B**) and late (**C**) declining phase. In the last two microscope pictures (x400) there are clear evidence of cell suffering, lysis and formation of aggregations of cell debris that are also common facets of bloom demise in open waters. Scale bar depicts 5 µm.

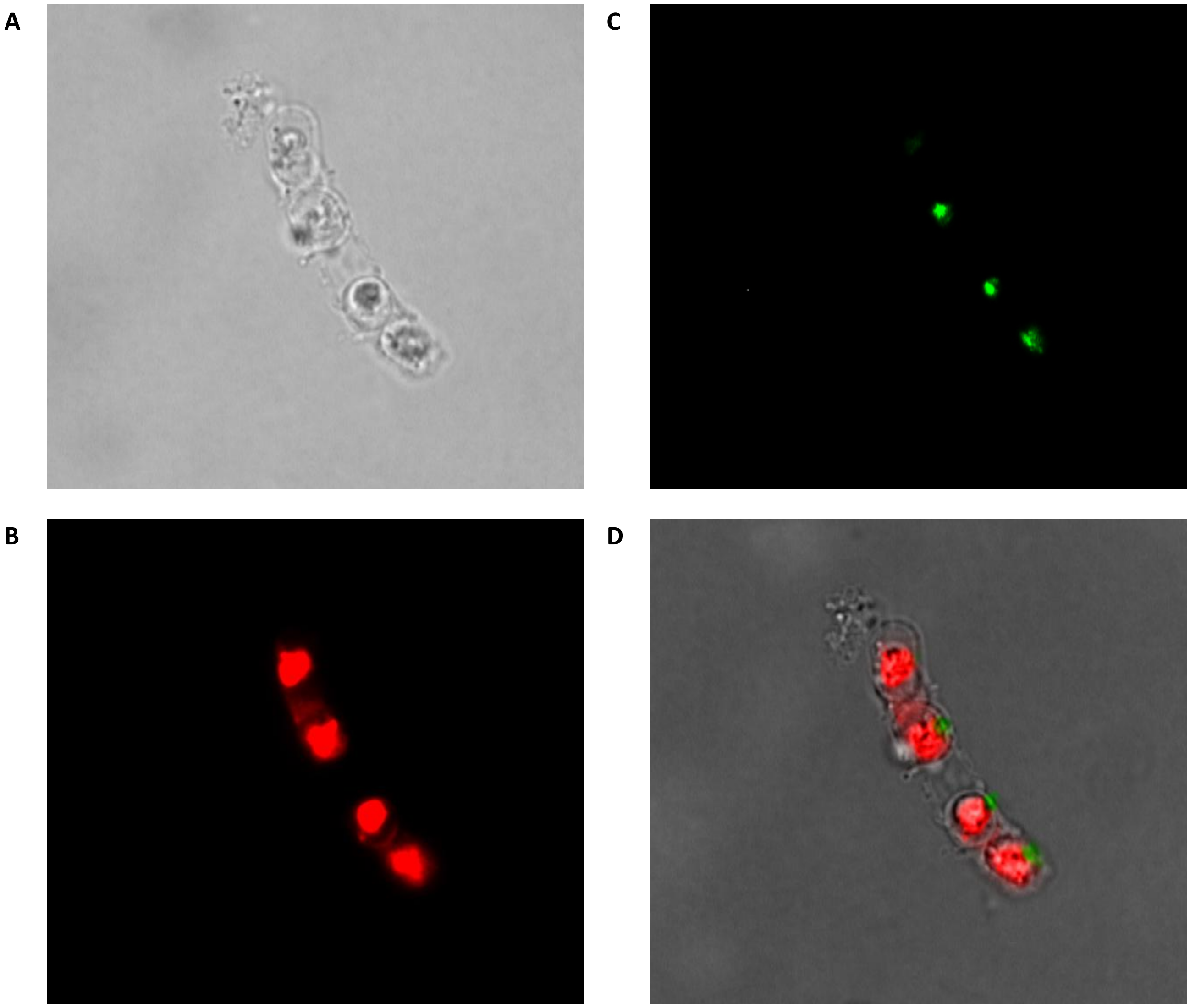

Supplementary Figure 14. Representative images of *S. marinoi* cells after treatment with CHOS. Cells are stained by Annexin -FITC for phosphatidylserine (PTS) externalization. (**A**) clear, (**B**) FITC fluorescence, (**C**) chlorophyll autofluorescence,(**D**)merge.

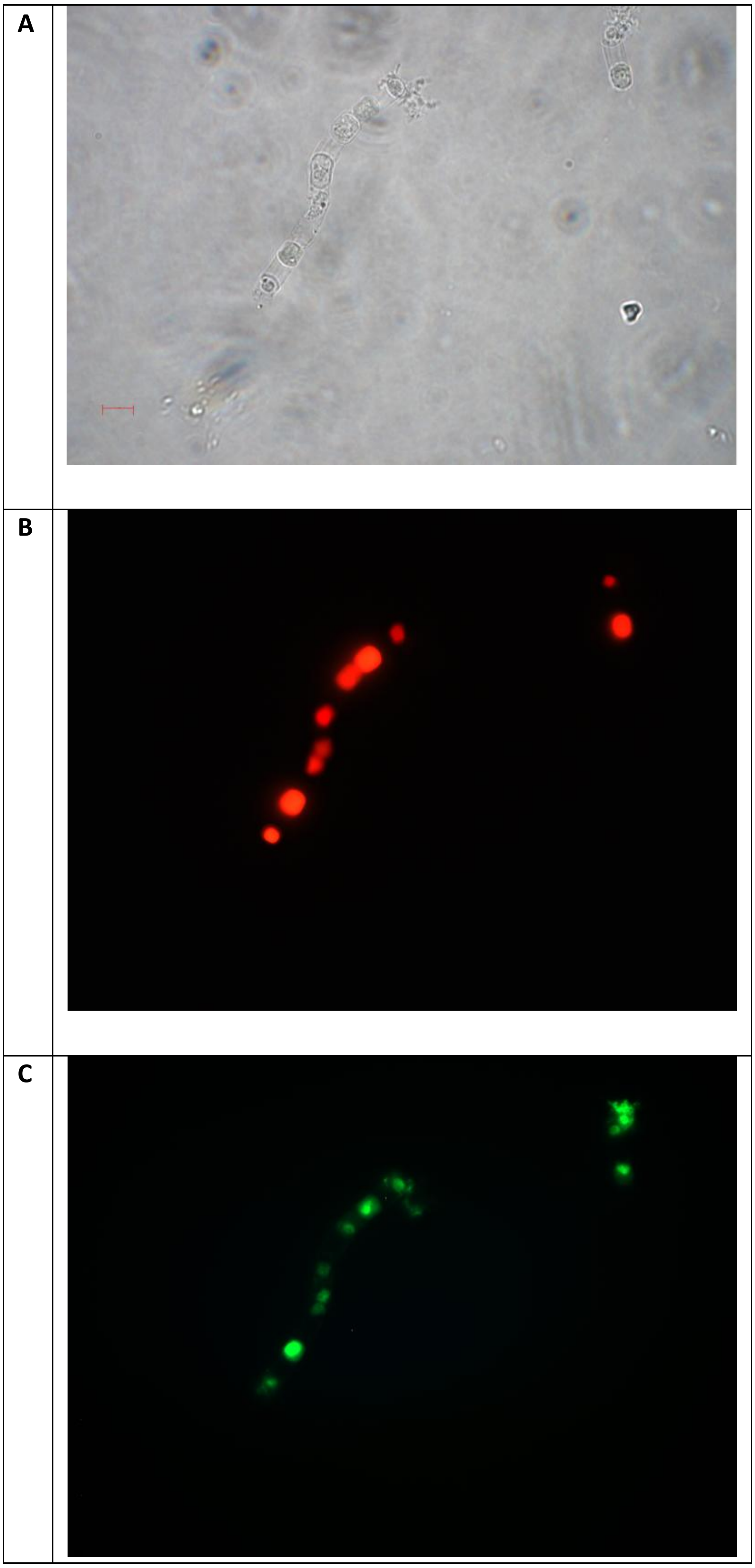

Supplementary Figure 15. Representative images of *S. marinoi* cells after treatment with CHOS. Cells are stained by TUNEL for DNA fragmentation. (**A**) clear, (**B**) chlorophyll autofluorescence and (**C**) TUNEL fluorescence. Scale bar depicts 10 µm.

Supplementary Table 1. NMR Spectroscopic data (600 MHz, CD_3OD) of sterol sulfates

	Dihydro-brassicasterol sulfate (**DHBS**)		β-Sitosterol sulfate (**βSITS**)	Cholesterol sulfate (**CHOS**)
position	δ_C, type	δ_H multeplicity (*J* in Hz)	δ_H multeplicity (*J* in Hz)	δ_H multeplicity (*J* in Hz)
1	38.5	1.94, m		
		1.15, m		
2	29.3	2.09, m		
		1.67, m		
3	79.8	4.16, m	4.16, m	4.16, m
4	40.3	2.57, ddd (*13.0*, *5.1*, *2.2*)	2.57, ddd (*13.0*, *4.7*, *2.2*)	2.57, ddd (*13.0*, *5.1*, *2.2*)
		2.37, bt (*13.0*, *13.0*, 2.2)	2.37, bt (*13.0*, *13.0*, 2.2)	2.37, bt (*13.0*, *13.0*, 2.2)
5	141.5	-		
6	123.1	5.42, m (W/2 = *9.7*)	5.42, m (W/2 = *9.7*)	5.42, m (W/2 = *9.7*)
7	33.1	2.04, m		
		1.60, m		
8	33.4	1.51, m		
9	51.6	1.00, m		
10	35.5			
11	22.2	1.53, m		
12	41.2	2.09, m		
		1.22, m		
13	42.2	-		
14	58.2[a]	1.06[b], m		
15	25.2	1.65, m		
		1.16, m		
16	29.2	1.89, m		
		1.33, m		
17	57.3[a]	1.18[b], m		
18	12.3	0.75, s	0.75, s	0.75, s
19	19.8	1.07, s	1.07, s	1.06, s
20	37.5	1.41, m		
21	19.4	0.98, d (*6.7*)	0.99, d (*6.7*)	0.98, d (*6.7*)
22	34.7[c]	1.48[d], m		
		1.02, m		
23	31.8[c]	1.46[d], m		
		1.02, m		
24	40.3	1.25, m		
25	32.4	1.63, m		
26	16.0	0.83, d (*6.7*)	0.87, d (*6.7*)	0.91, d (*6.7*)
27	20.8	0.91, d (*6.7*)	0.89, d (*6.7*)	0.91, d (*6.7*)
28	18.0	0.84, d (*6.7*)		-
29	-	-	0.91, t (*7.2*)	-

Supplementary Table 2. Putative sulfotransferase sequence from *S. marinoi* transcript analysis

Transcript ID	Sequence
**TR14735i7**	GGGTGGATTAGTTAGCAAGGAAGACTTTGCTGCGGCTCTTCGTGGACATCAGGCTGCTGTAGACGCAACGAA<br>GAGTCCCCAGAGGGAGGAAGCAGAAATAGCGTTGAGTCAGCTGAGGGAGGCAGCAGAAGCAGCTCGAAAG<br>GATAGAGTAAAATAAACTGCATCTCTGTATCTCAAGCAAGAACAGAATCCTATCCAATTTATTGCGCTCAGATG<br>CGACGACTTCAATACATATGCTCTTGCTCTCCCGGTGGATATGTATCGCTCACTTCTCCACCACACTCAAAAATA<br>CCTCTCTACAATCCTCATAACAATCACCTTTCAACACATTGGAAATCTTGGAACGATATTCCTCTCTCTCCACCCA<br>GTCATTGTATGCAACCATCAATTCCGACGACTCTCCCTCCTCTTTCCCACCTCCACTATCATTCGTGCTCCTATTC<br>AATAACAACCTCCTCCCATCGCCCGTGACCCCCTTCCTCAAAAACTGAAATCCATTCAGCCACCCAACCGACTTG<br>GGCTGAAATTTCTCTATATTATTCTTCATCGAACTGAACCCAAATGATGGTAATATCTCTTGCTCAAGCACACGT<br>GATGGGATGTGTGTAAGGTTTAAGAAGGAGATGATTCGAAGTACTTCGGTGCGGAGGTTGGATTTCATCTTTT<br>CGTAGGATAGAAGGAGCAGTGGCTGATCTGTAACACTATTGCTATTGCTTTCATCATCACCAATGTCAGCATGG<br>CCGCAAGAAGTAGTTGTGTCATACAAATTATCAGCAAACCCACCAGCATAACTAATCAAATGATGCAAATTGCT<br>TCCAAATGGAAGCTTGCCATCCATCCAATCTTGAAGAAACTTTCAAATGTATCGGTATAGGTGCCCTTTTTTTTA<br>TTCGATAAGTGATGGTAGAATGAGGCTACGACATCTATTTATTTCTTGTAACATAGATGAACTTTCCGCATTGA<br>GGAGGACGCTCTTTGTGAATGGTGCTGTCGTTATCAGCTGTTGATATTGTATTGCCATCGCCCCCAACATCGTC<br>GTCTTTTTGTTGTGTGCCACTCCTCGCTTCTCTTTGCTTGGGCAACATATCCCAACGTAAATGAGTATTAAACAC<br>CCTTCGTCCTAATCGATCATGATTCTCTCGTACCGAATCCGCCAATACCTTCGTGCTATT
**TR5935i1**	CTTACATTTATAGTTTGTTGGAGCATACTGAATTCTACAAAATGATCCATTCGCGTGGCACACCCAACGGCTGC<br>AACTTTTCTTTGTACAACGATTCCAATGCAGCAAGCTGTTCAGTTGAAAACGCAAGTCTACTACCATCAGCACTT<br>CCAACCTCTCCCTGACGAACATGTGCTCTTCGAAGAACACCAAGCCATCTCATGAACCGCAGTCCCATTTCTTGT<br>TCATCTGCTAATTTCATCCGGTCAAAGCTTGTAACATCCATAACATTTTGAATCTTGGTGGGAGTGACATCATCC<br>ATTCCAATAAATGCTGCAATTTGCTCAATCACATAACTTGGATTACGCTTCAGTTCTTCAAATCTCACCAACAAA<br>ACATCTGGATCATCCGCCCGCTTAAACCATGACGCCACATGATCAAAGTAGGAGCCATTCTCCACATTCCCATC<br>CAACCAATCCTGGACAAAGTCATCCCAACCTCCCTCAAATGTCATATCTTCCGTCCCATATGAAACCCCATTCCA<br>AAACATTGATTCCAATGTGAAATCAGTAGATCGGA
**TR11343i2**	TTTTTTTTTTGATTACAATAAGCTAGAAGCAACTTTAAGTATGTAAGCTACAATAATTATTGTTTTTGCTTCCTCG<br>TACATATTTGTTATTCATGCGGAGCCAAGCTTTCCCTTCTCTTACGTAGCGAAGGCATCTGGTCAAGAGTGACG<br>CTGGATCTTCGCCTCAAAATGCTCTTACGGACTCCTTGCTCAACAACTTCTACCTCCTCCTCCGTAGCACTTAAGT<br>CCGCAATTGCTGCTGCAAATTCGTCATAGCTAGCATGACCAGTCAGAGGAGTCATTCTTTCCAACCATCGATCC<br>TCCATCCATTCAATTGTCTTTTCTGTAAGCACAGTATCTTTCTTTTTACCATCTATACTTCTCACTTTGGCAGCAGG<br>CTCCATAACTCTCAAGGCACGGCCAAGTTCTCTGCCCTTTTCTGTGATGAAGTGATCATCAAATGCGTCCACATG<br>CTTTACCATTTGGTCACGAGATATCAAGAACGCCACCTTAGAATATAAAGCTTCATCTGCATCATCTATGCCTAA<br>AAACTCGGCAATCATGGGCAAATGGTTGAGATATGCAGGTCCATCTGCCACCAATGCTTCGTAGACAAGAATC<br>TTTACAGATGGGTCGTTCCTTGCCGACCAAATCTCTGCGTACATCTCCCAGATATTCATACCAAAGCAACATTCC<br>TCGCCGAAAATATCTGCTCCCTTATTTGCAATATACTCATTTGCATCTTCATACTCAGCGTAGCCAGGCCTCCCCT<br>TTGCCTTTTGAAATGCAAACCAACTCATCAACGTAGCTTCTGGATCACGAACAATACACAAGTATTTGCCTCCCG<br>GATTGACAGGCGATAAAAGTTGATGTGATTTGAAGATACGAGGACGAGCAACTTGATCATCATCTAAATCTTG<br>ACCCAGGTCATGAGCAAACTCTATCCACGGCTGCCGGATGGTGATATCGTCAAAGTCATATCCACCAGCCCGA<br>GTTCGCAACTGTTCACAGCAATTCTGCGTCCAAGTAGTACCCGTTTTGGGGAAGGAAAGAACAACCACGTCGG<br>TTGGACGAAGCCGGAATGTCTCGCCGATGCGCTTGGACTCTGGTGAAATATGTGGCATGACCTTGTAGGGCTT<br>GGTGCCCCAGTTCGGCCAGCGGTTCACATCGCTGATGTTCCCTTCTTTCTTAGTGGATTCCATGCTGCTGTTGTC<br>TACTTACTGAAGAGTTGCGTGTTGTGTGTAGGAAAGCAAGGTGAGA
**TR8670i1**	CTTCCATCTGTAGTTGAACTGTGCTTAGGTATGAAGTAGATCGGGACAGCGTATTGTACAGTCGGTGATTTACC<br>GGTGAAAGTATAAGAAGTAGATCAAATAACATGCACCAATAATGTTTTGCACAGTAACGAGCCAAGCAGGACT<br>GCTACTTCGATTCTATCTGATCATCACTATTTATGATACAGCTCACCAAGAAACTCCAACTCCTCCTTACTAAACA<br>CCTCCTCCAGCCTTTCTTGCAACTTGTCGGCCTGATCCACGCTCATCAACTCCCTCCAACCTCCCGTACTCCCCCG<br>TCGAATAAATGGTTGATACCCTGTCCTATCGCTACACCACCGAGTTGGGTCCTTCTTCATACTATCCAATGAGGA

	ATGATCTAGCACTAGGTTTAATAAACGTTCATTGTCTGCGAGGAGTTTGGCAGGGTAAATATCCTCATTTAAGA<br>ATTTGGCAATTTGTAATACGTATTCTCGTGGGTTGGCACGTCCATTCTCATATCTTAGAAACAAGACATTGGGAT<br>CATCTTTGTGGTTCAGCGCTTTTCGTGTGAAATCGAAATAGTCACCATGATCGACGTGACCATTGAGAAAGAGA<br>TTGAAGTAGGTATCGAAGTGTCCATCGGCGAAATTGTAGTGTCGGGGAAATCCAACCGTGTGATGATAGAATG<br>ATACCACGCAGTCTTTGGGATTGCGCACAACAAAGATGTATTTTCCTTTTTTATTTTGAGGTACCATGTCATACG<br>GGAGATGGGTTTTGATGAGTCGATATCCATCTTTGATTGTTGCATTCGTTTTTACAAATTCTTTCCCCACCTCTTC<br>TAGATGGGGAAAGACCACGTCGAGGCGTTCATCTGGAGAGAGGGGCACGCCGTTATTGAGGATTAGATAGAT<br>TATATGTTGGGTCCATGTCGTGCCACACTTTGGATATGTTACGATGAATAAATCTTCATCTTTCGCTTCATATTC<br>GAGACCGCTGCGAAACCCATCGAGAGGGAACCCCTTTGCCAGCTTATAGCCGTCATGGTCGGTGTAGGTAGG<br>GGTCTTTTCGGAGGCCATGGTCAAAAGAATGATGTCGAAAGATG

Supplementary Table 3. Predicted SULT sequences of *S. marinoi*

Transcript ID	Protein	Predicted sequence
**TR14735i7**	***SMAR*- 1**	NSTKVLADSVRENHDRLGRRVFNTHLRWDMLPKQREARSGTQQKDDDVGAMAISTADNDSNVRK<br>ERPQCGKFIYVTRNQIDVVASFYHHLSNQIEGTYTDTFETFLRDWMDGKIPVGSSLHHLIGFAGGFAD<br>NLYDTSCDHDIGDDENNSVTDQPLLLLSYEKMKSNLRTEVLRIISFLNLTHIPSRVLEQEILPSFGFSSMK<br>NNIEKFQPKSVGWLNGFQFLRKGVTGDGRRLLLNRSTNDSGGGKEEGESSELMVAYNDWVEREEY<br>RSKISNVLKGDCYEDCREVFLSVVEKAIHIHRESKSICIEVVASERNKLDRILFLLEIQRCSLFYSILSSCFCC<br>LPQLTQRYFCFLPLGTLRCVYSSLMSTKSRSKVFLANST
**TR5935i1**	**SMAR- 2**	RSTDFTLESMFWNGVSYGTEDMTFEGGWDDFVQDWLDGNVENGSYFDHVASWFKRADDPDVLL<br>VRFEELKRNPSYVIEQIAAFIGMDDVTPTKIQNVMDVTSFDRMKLADEQEMGLRFMRWLGVLRRA<br>HVRQGEVGSADGSRLAFSTEQLAALESLYKEKLQPLGVPREWIIL-NSVCSNKL-M-
**TR11343i2**	**SMAR- 3**	LTLLSYTQHATLQVDNSSMESTKKEGNISDVNRWPNWGTKPYKVMPHISPESKRIGETFRLRPTDVV<br>VLSFPKTGTTWTQNCCEQLRTRAGGYDFDDITIRQPWIEFAHDLGQDLDDDQVARPRIFKSHQLLSP<br>VNPGGKYLCIVRDPEATLMSWFAFQKAKGRPGYAEYEDANEYIANKGADIFGEECCFGMNIWEMY<br>AEIWSARNDPSVKILVYEALVADGPAYLNHLPMIAEFLGIDDADEALYSKVAFLISRDQMVKHVDAFD<br>DHFITEKGRELGRALRVMEPAAKVRSIDGKKKDTVLTEKTIEWMEDRWLERMTPLTGHASYDEFAA<br>AIADLSATEEEVEVVEQGVRKSILRRRSSVTLDQMPSLRKRRESLAPHE-QICTRKQKQ-LL-<br>LTYLKLLLAYCNQKK
**TR8670i1**	**SMAR- 4**	SFDIILLTMASEKTPTYTDHDGYKLAKGFPLDGFRSGLEYEAKDEDLFIVTYPKCGTTWTQHIIYLILNN<br>GVPLSPDERLDVVFPHLEEVGKEFVKTNATIKDGYRLIKTHLPYDMVPQNKKGKYIFVVRNPKDCVVS<br>FYHHTVGFPRHYNFADGHFDTYFNLFLNGHVDHGDYFDFTRKALNHKDDPNVLFLRYENGRANPRE<br>YVLQIAKFLNEDIYPAKLLADNERLLNLVLDHSSLDSMKKDPTRWCSDRTGYQPFIRRGSTGGWREL<br>MSVDQADKLQERLEEVFSKEELEFLGELYHK---SDRIEVAVLLGSLLCKTLLVHVI-<br>STSYTFTGKSPTVQYAVPIYFIPKHSSTTDG

Supplementary Table 4. Domain characterization of *S. marinoi* putative SULTs sequences reported in Supplementary Table 3

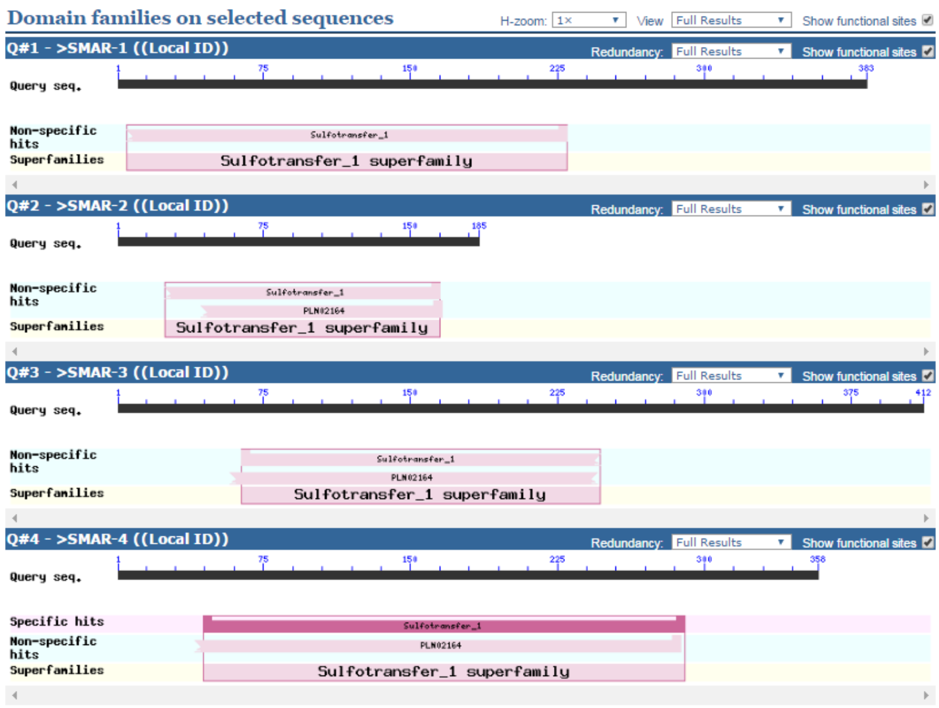

Query	Hit type	PSSM-ID	From	To	E-Value	Bitscore	Accession	Short name	Incomplete	Superfamily
Q#1 - >SMAR-1	superfamily	304426	5	230	2.69325e-15	73.5338	cl21551	Sulfotransfer_1 superfamily	N	-
Q#2 - >SMAR-2	superfamily	304426	25	165	3.02443e-18	78.1562	cl21551	Sulfotransfer_1 superfamily	N	-
Q#3 - >SMAR-3	superfamily	304426	64	247	2.97374e-16	76.6154	cl21551	Sulfotransfer_1 superfamily	C	-
Q#4 - >SMAR-4	specific	279075	44	290	5.34283e-52	171.76	pfam00685	Sulfotransfer_1	-	cl21551

Supplementary Table 5. Reference sequence number of proteins used for phylogenetic analysis

Organism	Protein reference number
Arabidopsis lyrata	**XP_002890763.1**
Arabidopsis thaliana	**OAP14364.1 SOT7**
Brassica rapa	**XP_013723193.1**
Camelina sativa	**XP_010425175.1**
Capsella rubella	**XP_006293532.1**
Chrysochromulina_sp.	**KOO25185.1**
Crocosphaera watsonii	**WP_007308169.1**
Cyanothece sp. ATCC51142	**ACB53617.1**
Cyanothece sp.CCY0110	**WP_008278423.1**
Cyanothece sp. PCC7424	**WP_012598684.1**
Cyanothece sp.PCC8801	**ACK66184.1**
Daucus carota	**XP_017233697.1**
Emiliana hyxley	**XP_005757376.1**
Fragilariopsis cylindrus	**OEU18550.1**
Homo sapience 1C	**AAC95519.1**
Homo sapiens 1E	**AAH27956.1**
Homo sapiens 2B1A	**1Q1Z_A**
Homo sapiens 2B1B	**NP_004596.2**
Micromonas commoda	**XP_002509199.1**
Nannochloropsis gaditana	**EWM21324.1**
Phaeodactylum tricornutum	**XP_002179849.1**
Skeletonema marinoi Fe7-1	**MMETSP1039-20121108|3029_1**
Skeletonema marinoi Fe7-2	**MMETSP1039-20121108|3249**
Skeletonema marinoi Fe7-3	**MMETSP1039-20121108|14798**
Thalassiosira oceanica	**EJK47508.1**
Thalassiosira pseudonana-1	**XP_002290864.1**
Thalassiosira pseudonana-2	**XP_002292441.1**
Trichodesmium_erythraeum	**WP_011609991.1**

Supplementary Table 6. Multiple ClustalW alignment of protein sequences containing the four SULT conserved regions and the acceptor site (AC). Protein sequences are the same of Supplementary Table 3.

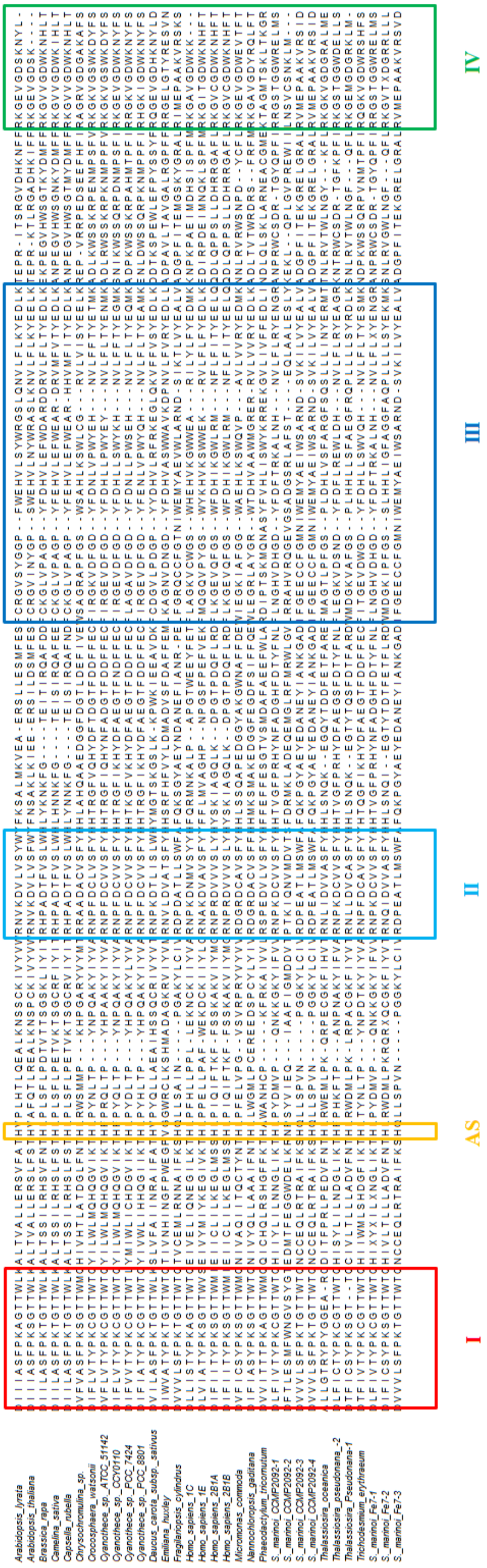

Supplementary Table 7. **Analysis report of *Skeletonema marinoi de novo* transcriptome assembly**: **number and quality of paired-end reads obtained for each sequenced sample**.

Sample	Reads	% of ≥Q30 Bases (PF)	Mean Quality Score
1	62,156,844	90.57	35.35
2	57,766,124	92.35	35.82
3	55,414,624	90.34	35.28

Analysis was carried out by a paired-end sequencing. Short reads are obtained from ends of DNA fragments for ultra high-throughput sequencing. Prior to further analysis, a quality check was performed on the sequencing data. The high quality reads from all the samples were joined and then used as input to perform transcriptome assembly by Trinity. The raw assembled transcriptome included about 51 Mbp in 56931 transcripts grouped in 43376 genes. The mean GC content was 45.24%. The average and median contig length were 911 bp and 546 bp, respectively. The N50 was 1467 bp.

Supplementary Table 8. **Analysis report of *Skeletonema marinoi de novo* transcriptome assembly**: **gene number expressed at least with 2 FPKM** (Fragments Per Kilobase of exon per Million fragments mapped) **in each sequenced sample.**

Sample	Num_features with at least 2 FPKM
1	18205
2	17958
3	17809

Supplementary methods

Bioassay-guided fractionation

Biomass was recovered at the first point of declining phase, extracted and fractionated on polystyrene–divinyl benzene solid phase columns (CHROMABOND® HR-X). Organic constituents were separated on the basis of polarity and molecular weight in 5 fractions with decreasing polarity from water to dichloromethane (H_2O, CH_3OH:H_2O, CH_3CN:H_2O, CH_3CN, CH_2Cl_2:CH_3OH). These fractions were tested in 24-wells assay on *S. marinoi* cells. The activity was found to be conserved in the fraction eluted with CH_3CN:H_2O at concentration of 20 µg mL^{-1}. The bioactive mixture was then fractionated by HPLC on a reversed phase semipreparative column and the fractions were collected every two minutes. All the fractions were tested again and we obtained a single bioactive sample at a concentration slightly above 10 µg mL^{-1}. These fraction was further purified in the last HPLC step to yield cholesterol sulfate (CHOS), dihydrobrassicasterol sulfate (DHBS) and β-sitosterol sulfate (βSITS).

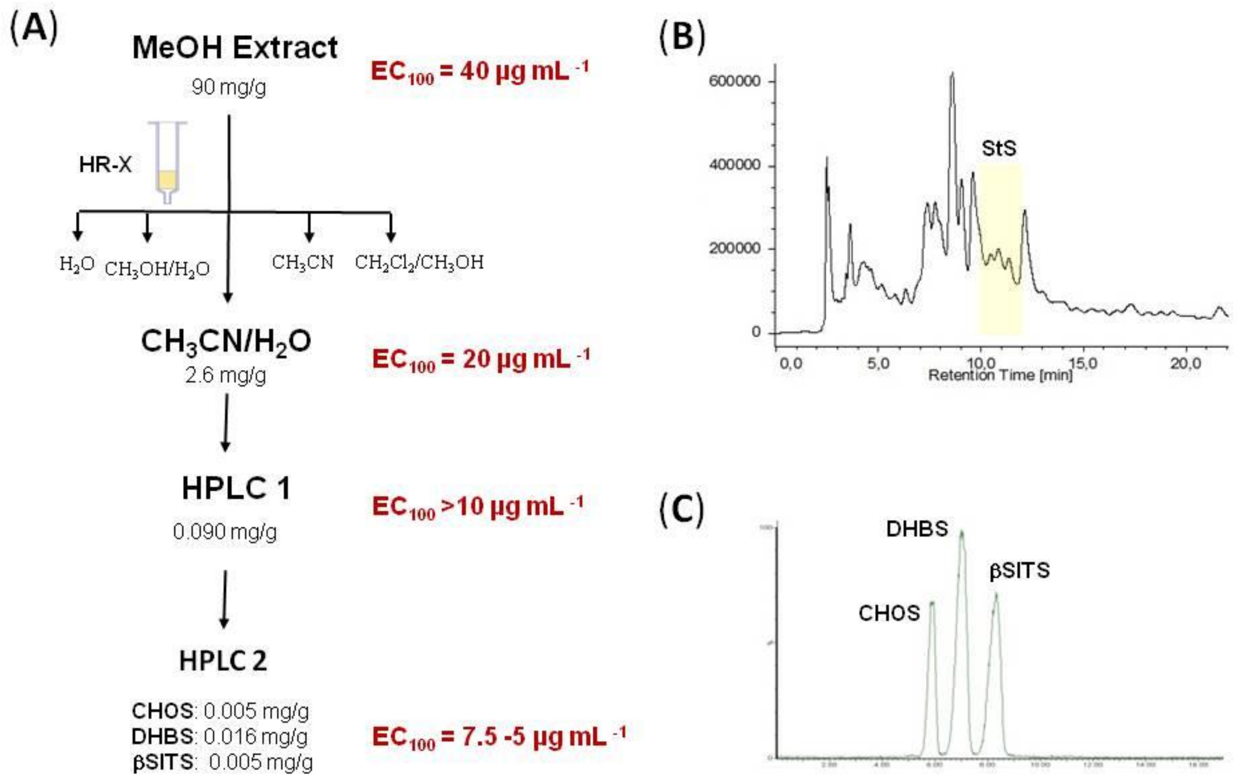

Supplementary Figure 16. Scheme of protocol of isolation of sterols sulfates. (**a**) Bioassay-guided fractionation. The toxic doses (100% mortality in 48 hours) for each step of purification are reported in red. Below each step is reported the average quantity of material from 1g of *S. marinoi*. (**b**) and (**c**) chromatographic profiles of the first and second HPLC steps.

Complete TUNEL assay protocol

The complete protocol consists of three phases that can be described as follows:

1) Fixation of cells: 10 mL of *S. marinoi* cultures at a concentration of about $1\cdot 10^6$ cells mL^{-1} for each sample were centrifuged and resuspended in 1 mL of filtered sea water with 2% formaldehyde. The cells were left at 4 ° C for 20 min.

2) Permeabilization of external cell membranes: cells are centrifuged (each sample now has the concentration of $1\cdot 10^7$ cell mL^{-1}) and washed with PBS at pH 7.5 containing 5 mM $MgCl_2$, which corresponds to the concentration required for maximum activity of Tdt enzyme and DNase enzyme used as a positive control. Then each sample is suspended in 200 µL of permeabilizing solution: 3% Triton X-100 in 0.1% sodium citrate on ice for 15 min. Cells were again washed with PBS. Positive controls are incubated 10 min with 10 µg mL^{-1} DNAse I (Roche) solution in PBS containing 1 mg mL^{-1} of BSA at room temperature. Subsequently cells were pelleted and washed with PBS.

3) Incubation with the reaction mix (enzyme + labeled nucleotides in reaction buffer): the free 3'-OH of DNA strand breaks produced during the process of apoptosis were labeled with green- fluorescing fluorescein labels incorporated in modified nucleotide polymers in an enzymatic reaction. Cells were labeled according to the manufacturer's instructions (Roche Diagnostics GmbH) and analyzed by epifluorescence microscopy (Carl Zeiss) using a 515/565 BP (for only green fluorescence), a 525/50 BP (for green and red fluorescence) and a LP615 (for only red fluorescence) emission filter and a coupled-device camera interfaced with Axio Vision acquisition/image analysis software (version 4.8). All assays were repeated in triplicate.
